# Risk factors for obstetric anal sphincter injury recurrence: A systematic review and meta‐analysis

**DOI:** 10.1002/ijgo.13950

**Published:** 2021-10-20

**Authors:** Marta Barba, Davide P. Bernasconi, Stefano Manodoro, Matteo Frigerio

**Affiliations:** ^1^ Department of Obstetrics and Gynecology University Milano‐Bicocca Monza Italy; ^2^ School of Medicine and Surgery University Milano‐Bicocca Monza Italy; ^3^ Department of Obstetrics and Gynecology ASST Santi Paolo e Carlo, San Paolo Hospital Milano Italy; ^4^ Department of Obstetrics and Gynecology ASST Monza, San Gerardo Hospital Monza Italy

**Keywords:** anal incontinence, anal sphincter injury, meta‐analysis, perineal trauma, systematic review

## Abstract

**Background:**

Women with previous obstetric anal sphincter injuries (OASIs) are at a higher risk of recurrence in the subsequent pregnancy, which may lead to the development or worsening of anal incontinence. Due to a lack of evidence, few recommendations can be made about the factors that may affect the risk of OASI recurrence.

**Objective:**

We sought to conduct a systematic review and meta‐analysis to investigate potential risk factors for recurrent OASIs.

**Search strategy:**

Studies up to May 2019 were identified from PubMed, Scopus, Cochrane Library, and ISI Web of Science.

**Selection criteria:**

Studies assessing the impact of risk factors on OASI recurrence in subsequent pregnancies were included. Reviews, letters to the editor, conference abstracts, book chapters, guidelines, Cochrane reviews, and expert opinions were excluded.

**Data collection and analysis:**

Data were extracted by two independent reviewers. Odds ratio and standardized mean difference were chosen as effect measures. Pooled estimates were calculated using the random‐effects model.

**Main results:**

The meta‐analysis showed that maternal age, gestational age, occiput posterior presentation, oxytocin augmentation, operative delivery, and shoulder dystocia were associated with the risk of recurrent OASIs in the subsequent delivery.

**Conclusion:**

Prenatal and intrapartum risk factors are associated with recurrence of OASI.

PROSPERO registration no. CRD42020178125.

## Abstract 2

Maternal age, gestational age, occiput posterior presentation, oxytocin augmentation, operative delivery, and shoulder dystocia are associated with obstetric anal sphincter injury recurrence.

## 1 INTRODUCTION

Anal sphincter injury during childbirth is a major cause of anal incontinence. Rates of obstetric anal sphincter injuries (OASIs) are not well defined, ranging widely from 0.6% to 19.3%.^1^, ^2^ In a large cohort study in Denmark, OASIs have been estimated to occur in 3.6% of vaginal deliveries.^3^ However, the real incidence of OASIs has probably been underestimated. Currently, we are facing a trend towards a rise in the rates of OASIs. An increase in OASI rates has been reported in the UK, Australia, Scandinavia, and the USA.^1^, ^4^, ^5^, ^6^ OASIs are associated with short‐ and long‐term morbidity, which can have psychological effects and seriously affect quality of life. Overall outcomes after primary repair are encouraging, with 62% of women asymptomatic after a primary repair.^7^ However, data show a worsening of anal incontinence after a subsequent vaginal delivery in 17%–24% of women with previous OASIs.^8^ Moreover, the rate of recurrent anal sphincter injuries is increased compared with primary events by up to 13.4%^9^. Finally, it is unclear whether cesarean section is effective in preventing the development of anal incontinence in women with previous OASIs. As a consequence, it is difficult to properly counsel women with previous OASIs about the risk of anal continence worsening after a subsequent delivery. In particular, few recommendations can be made about the mode of delivery and factors that may affect the risk of OASI recurrence. The lack of systematic reviews or meta‐analysis affects the counseling that can be given by caregivers, failing to address patients’ concerns about the risks of recurrence, and even acting as a deterrent to further childbirth. A better understanding of the factors that contribute to recurrent OASIs would enable women and clinicians to make better informed decisions about the preferred method of subsequent deliveries.

The aim of the present systematic review was to investigate the risk factors for recurrent OASIs, describing their impact in terms of significance and strength of association.

## 2 MATERIALS AND METHODS

### 2.1 Study protocol

The present systematic review was conducted and reported according to both the PRISMA Statement for Reporting Systematic Reviews and Meta‐Analyses^10^ and the Meta‐Analysis of Observational Studies in Epidemiology guidelines (Files S1 and S2).^11^ Study objectives, eligibility criteria, outcome definitions, search strategy, data extraction process, statistical analyses, and method of study quality assessment were all defined in a protocol. All investigators were experienced in systematic reviews.^12^

### 2.2 Eligibility criteria and outcomes definition

Studies assessing the impact of risk factors on OASI recurrence in the subsequent pregnancies were included. Reviews, letters to the editor, conference abstracts, book chapters, guidelines, Cochrane reviews, and expert opinions were excluded. We considered outcomes variables investigated as potential risk factors for OASI recurrence in the subsequent pregnancies.

### 2.3 Data source and literature search

To identify potentially eligible studies, we searched PubMed, Scopus, Cochrane Library, and ISI Web of Science (up to May 10, 2019), using EndNote x8 (Clarivate Analytics). No language restrictions were applied. We used a combination of keywords and text words represented by "OASIS", "anal sphincter injuries", "severe obstetrical tears", “third degree tears”, “fourth degree tears”, "subsequent pregnancies”, "future pregnancies", "recurrence", and "risk factors". An example of the complete search strategy used for the PubMed search is presented in Appendix S1. Two reviewers independently screened the titles and abstracts of the records that were retrieved through the database searches. We also performed a manual search to include additional relevant articles, using the reference lists of key articles published in English. Both reviewers independently recommended studies for the full‐text review. Full texts of records recommended by at least one reviewer were screened independently by the same two reviewers and assessed for inclusion in the systematic review. Disagreements between reviewers were solved by consensus.

### 2.4 Data extraction and study quality evaluation

Data were extracted using a piloted form specifically designed for capturing information on study characteristics (sample size, outcomes, and considered variables). Data on all variables investigated by the study as possible risk factors were collected. These included maternal characteristics, index delivery characteristics, subsequent pregnancy characteristics, neonatal characteristics, and others. For clinically relevant variables, such as episiotomy and instrumental delivery types, data were collected when available for subanalysis. Data for continuous variables were extracted as means and standard deviations; for categorical variables, data were extracted as absolute values. Data were extracted independently by two authors to ensure accuracy and consistency. Authors of excluded studies were emailed if we felt that potentially they may have unpublished data about OASI recurrence. We received some answers, but no new dataset was obtained. Two reviewers independently screened full texts of records included in the systematic review. The scale contained four items under the selection domain, one item under the comparability domain, and three items under the outcome domain. A star scoring system, from zero to nine stars, was used for the assessment of study quality, such that the highest quality studies were awarded one star per item, except for the comparability domain, for which two stars for a single item could be assigned. Disagreements between reviewers were solved by consensus.

### 2.5 Statistical analysis

For each risk factor of interest, pooling of results was carried out according to the random‐effects method of DerSimonian and Laird.^13^ For binary risk factors, the odds ratio was considered as the measure of effect, adding a correction factor of 0.5 to the event frequency of studies where no patient had the outcome in either one of the exposure groups.^14^ For numerical risk factors, studies applying categorical analysis were excluded due to heterogeneous cut‐offs used. For numerical risk factors, the standardized mean difference was chosen as the measure of effect. For studies reporting only median and range or interquartile range, the method of Wan et al.^15^ was used to approximate mean and standard deviation. I^2^ and τ^2^ indexes were used to quantify heterogeneity between studies and the null hypothesis that all studies share a common effect size was tested. For the meta‐analysis of risk factors where at least nine studies were available, a funnel plot was produced and the Egger test was performed.^16^ All analyses were performed using the R package (R Foundation for Statistical Computing, Vienna, Austria) “meta”.^17^ For risk factors considered in only one study and statistically significant, a narrative description was adopted.

## 3 RESULTS

### 3.1 Study assessment

**FIGURE 1:**
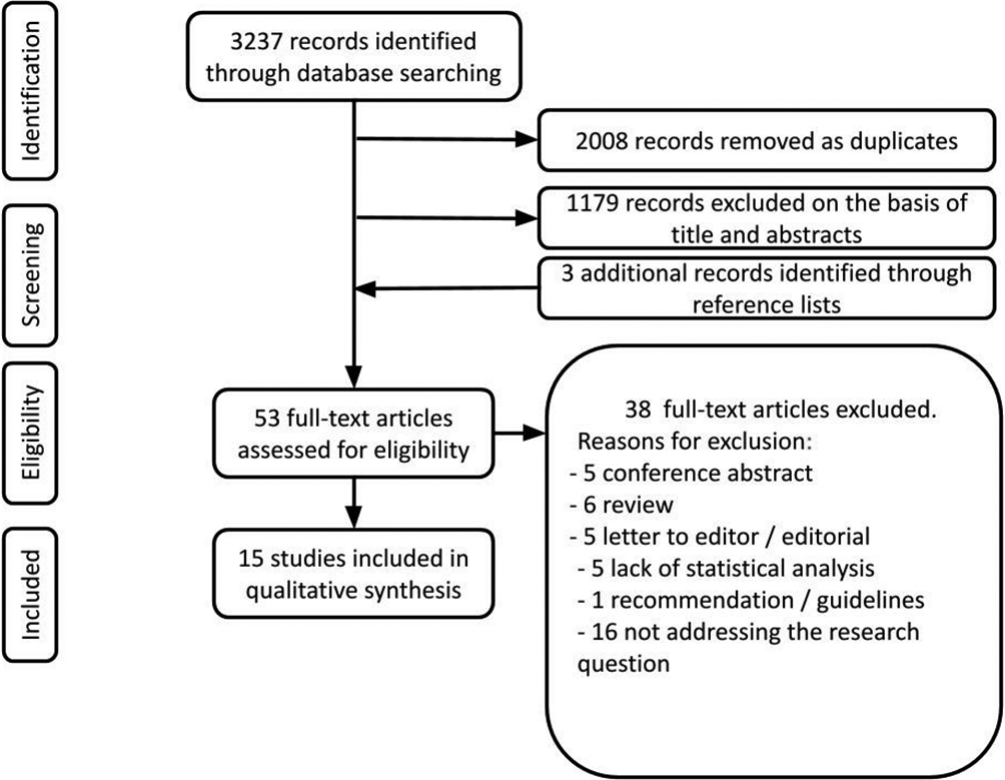
The electronic database search

**FIGURE 2:**
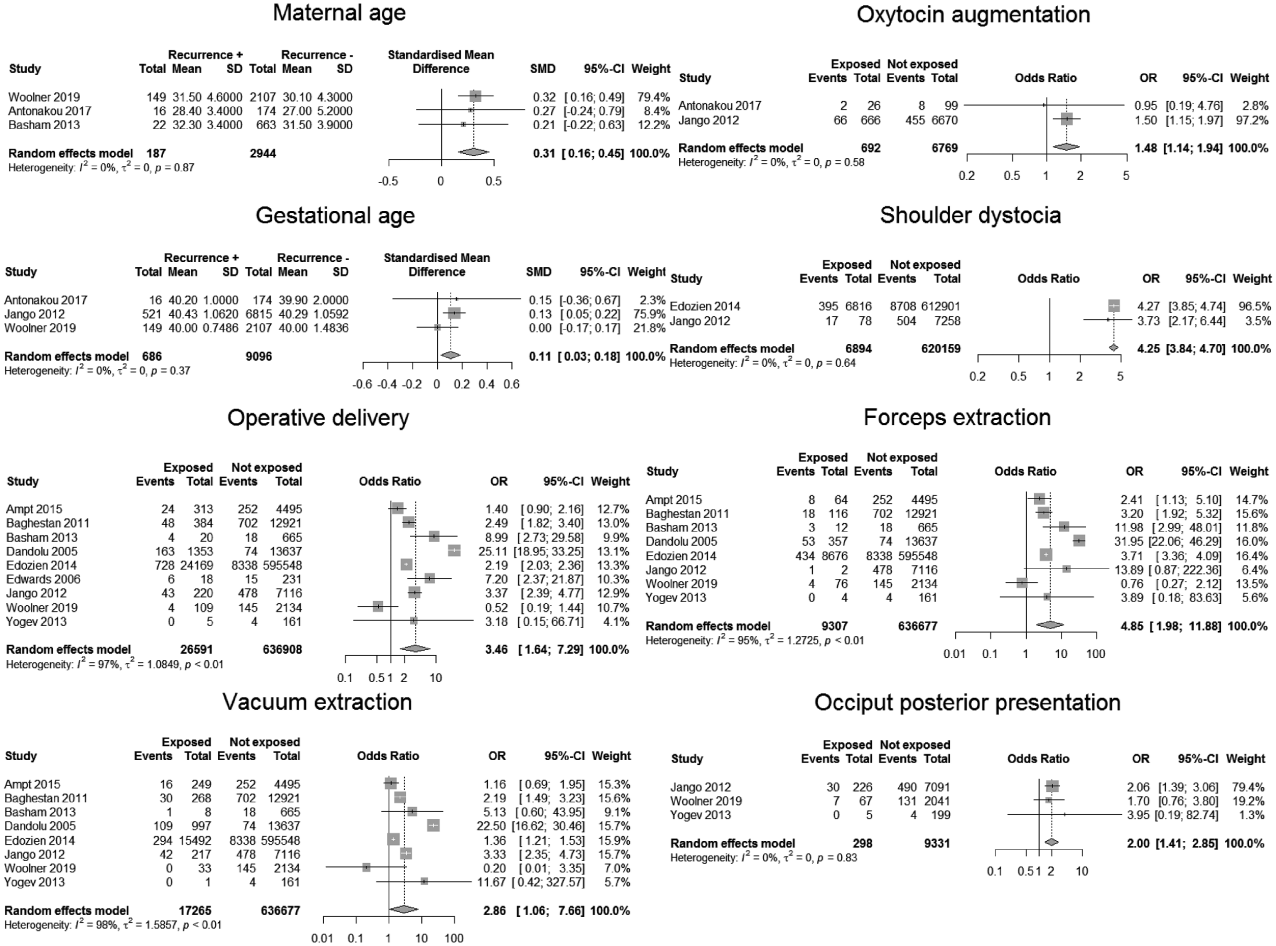
Forest plot for variables with significant correlation with obstetric anal sphincter injury recurrence

The electronic database search provided a total of 3237 results (Figure 1). After excluding duplicates, 1229 citations remained. Of those, 1179 were not relevant to the review based on title and abstract screening. Fifty‐three studies were considered for full‐text assessment, of which 38 were excluded for the following reasons: there were six reviews, five conference abstracts, five letters to the editors, and one guideline; 16 papers were excluded for not addressing the research question; five studies were excluded due to lack of statistical analysis. None was excluded for languages other than English. Overall, 15 studies met the inclusion criteria and were incorporated into the final assessment.^9^, ^18^, ^19^, ^20^, ^21^, ^22^, ^23^, ^24^, ^25^, ^26^, ^27^, ^28^, ^29^, ^30^, ^31^ The main characteristics of these studies are listed in Table 1. Different study designs resulted from the selection process, including register‐based and retrospective studies. The studies included were very heterogeneous clinically. All the risk factors proposed by the considered studies were analyzed for a total of 34 variables, grouped in five categories (see Table 1). A funnel plot and the Egger test were only possible for two index delivery characteristics (episiotomy and operative delivery; Fig. S1). Forest plots demonstrating significant associations are shown in Figure 2. The meta‐analysis not demonstrating significant associations is available in Figure S2.

**TABLE 1:** Studies characteristics

First author	Year	Reference	Country	Study design	Population (*n*)	rOASIs (*n*)	rOASIs (%)	Risk factors assessed				
								Maternal	Index delivery	Current pregnancy	Neonatal	Others
Ali	2014	9	Ireland	Retrospective	138	11	8.0	X			X	
Ampt	2015	18	Australia	Retrospective	6380	276	4.3	X	X	X	X	
Antonakou	2017	19	UK	Retrospective	11 191	603	5.4	X	X	X	X	
Baghestan	2012	20	Norway	Registry‐based	13 305	750	5.6	X		X	X	X
Basham	2013	21	USA	Retrospective	685	22	3.2	X	X	X	X	
Boggs	2014	22	Canada	Retrospective	9857	102	1.0			X	X	
Dandolu	2005	23	USA	Registry‐based	14 990	864	5.8	X		X		
Edozien	2014	24	UK	Retrospective	619 717	9103	1.5	X		X	X	
Edwards	2006	25	USA	Retrospective	271	6	2.2	X		X	X	
Jangö	2012	26	Denmark	Registry‐based	7336	521	7.1	X	X	X	X	
Lowder	2007	27	USA	Retrospective	1054	76	7.2			X	X	
Payne	1999	28	USA	Retrospective	178	19	10.7			X	X	
Spydslaug	2005	29	Norway	Registry‐based	9558	357	3.7	X				
Woolner	2019	30	UK	Registry‐based	2256	149	6.6	X		X	X	
Yogev	201r	31	Israel	Retrospective	166	4	2.4	X	X	X	X	

Note

Newcastle‐Ottawa scale quality evaluation and variables considered: maternal characteristics: age, ethnicity, social status, cigarette smoke, weight, parity; index delivery characteristics: epidural analgesia, operative delivery, grade of obstetric tear (third degree vs fourth degree), concomitant episiotomy, suture material, wound complications, neonatal weight, diabetes; current pregnancy characteristics: years since index pregnancy, hypertension, diabetes, gestational age, induction, oxytocin augmentation, epidural analgesia, labor total length, first stage length, second stage length, fetal head position, operative delivery, episiotomy, shoulder dystocia; neonatal characteristics: weight, difference in weight compared with index pregnancy, gender, cranial circumference; others: impact of father, maternal unit deliveries per year.

Abbreviation: rOASIs, recurrent obstetric anal sphincter injuries.

### 3.2 Maternal characteristics

Maternal characteristics included age, ethnicity, social status, cigarette smoke, weight, and parity. Age was considered by 11 studies.^9^, ^18^, ^19^, ^20^, ^21^, ^23^, ^24^, ^25^, ^26^, ^30^, ^31^ Data pooling was possible for three studies.^19^, ^21^, ^30^ The meta‐analysis showed that older women were significantly more likely to have OASI recurrence, with a standardized mean difference of 0.31 (confidence interval 0.16–0.45). Maternal body mass index was analyzed by five studies.^19^, ^21^, ^25^, ^26^, ^30^ Data pooling was available for three studies.^19^, ^21^, ^30^ No differences were found in recurrent sphincter tears according to body mass index. The role of ethnicity was evaluated by two studies.^24^, ^25^ However, data pooling was not possible due to a lack of data in one of them. According to Edozien et al.,^24^ Asian ethnicity represented a risk factor for recurrent OASI, with an adjusted odds ratio of 1.59 (confidence interval 1.48–1.71). Cigarette smoke was only considered by one study^30^ and no association was found with recurrent sphincter tears. Parity was not related to variations in OASI recurrence risk according to the only study available.^31^ Two studies considered social status as a possible risk factor^24^, ^30^; one of them reported an association between recurrent OASIs and living in the least deprived communities.^24^ However, data pooling was not possible due to heterogeneity.

### 3.3 Index delivery characteristics

The index delivery characteristics were: epidural analgesia, operative delivery, type of obstetric tear (third degree vs fourth degree), concomitant episiotomy, suture material, wound‐related complications, neonatal weight, diabetes. The grade of obstetric tear (third degree vs fourth degree) during the index delivery was considered by three papers.^18^, ^19^, ^21^ Data pooling failed to show any association with the risk of OASI recurrence. Episiotomy at the index delivery was evaluated by three studies.^18^, ^19^, ^21^ The meta‐analysis of the studies did not show any relationship with sphincter tears in the subsequent deliveries. The roles of epidural analgesia and operative delivery during the index pregnancy were evaluated by two of the above three studies^18^, ^19^; no association was found with the risk of OASI recurrence. Diabetes and neonatal weight in the index pregnancy were considered only by Ampt et al.,^18^ who found no relationship with the risk of sphincter damage in the subsequent deliveries. Suture materials and wound‐related complications were evaluated only by Basham et al.^21^; no association was found with the risk of recurrent OASIs.

### 3.4 Current pregnancy characteristics

Current pregnancy characteristics included: interval in years from the index pregnancy, hypertension, diabetes, gestational age, induction, oxytocin augmentation, epidural analgesia, labor total length, first stage length, second stage length, fetal head position, operative delivery, episiotomy, shoulder dystocia. Gestational age was considered by four studies.^18^, ^19^, ^26^, ^30^ Data pooling was possible for three studies^19^, ^26^, ^30^ and showed that patients with recurrent OASIs were at greater gestational age, with a standardized mean difference of 0.11 (confidence interval 0.03–0.18). The role of induction was investigated by six papers^18^, ^19^, ^22^, ^25^, ^26^, ^30^; four of them were available for meta‐analysis.^18^, ^19^, ^26^, ^30^ However, the meta‐analysis did not show any relationship with recurrent OASIs. On the converse, the use of oxytocin showed a positive association with recurrence of sphincter injury, with an odds ratio of 1.48 (confidence interval 1.14–1.94). Data pooling was possible with two^19^, ^26^ of the four considered studies.^19^, ^22^, ^26^, ^28^ Episiotomy was investigated by 11 studies.^18^, ^19^, ^21^, ^22^, ^23^, ^24^, ^25^, ^26^, ^27^, ^28^, ^30^ A meta‐analysis performed on 10 of them^18^, ^19^, ^21^, ^22^, ^23^, ^24^, ^25^, ^26^, ^27^, ^30^ did not show any association with the risk of OASI recurrence. A subanalysis on either median or mediolateral episiotomy was available for three^19^, ^25^, ^27^ and two studies,^18^, ^26^ respectively. None of them showed a significant impact on recurrence of anal sphincter tears. Operative delivery was considered by 11 studies.^18^, ^20^, ^21^, ^22^, ^23^, ^24^, ^25^, ^26^, ^28^, ^30^, ^31^ Data pooling was performed on eight of them^18^, ^20^, ^21^, ^23^, ^24^, ^25^, ^26^, ^30^, ^31^ and showed an association with recurrent anal sphincter injury, with an odds ratio of 3.46 (confidence interval 1.64–7.29). This remained significant for both forceps (odds ratio 4.85; confidence interval 1.98–11.88) and vacuum extraction (odds ratio 2.86; confidence interval 1.06–7.66). The role of epidural analgesia was evaluated by five studies.^18^, ^19^, ^22^, ^26^, ^28^ Data pooling was available for two of them^18^, ^19^; no association was found with the risk of OASI recurrence. Fetal head position was analyzed by four studies^26^, ^27^, ^30^, ^31^; data pooling was possible for three of them.^26^, ^30^, ^31^ The meta‐analysis showed an increased risk of recurrent OASIs with occiput posterior presentation, with an odds ratio of 2.0 (confidence interval 1.41–2.85). Shoulder dystocia was considered by three studies.^24^, ^26^, ^27^ Data pooling was possible for two of them^24^, ^26^ and showed that shoulder dystocia represented a risk factor for recurrent sphincter injuries, with an odds ratio of 4.25 (confidence interval 3.84–4.70). Three studies evaluated the length of labor,^19^, ^24^, ^31^ either cumulative^19^, ^24^, ^31^ or first stage^19^/second stage alone^19^, ^22^; data pooling was not possible due to different cut‐offs applied. Four papers evaluated the interval between the index delivery and the subsequent delivery.^18^, ^20^, ^24^, ^26^ However, data pooling was not possible as only one study reported the measure as a continuous variable.^26^ This last study identified a significantly longer inter‐delivery interval (2.9 vs 2.7 years; *P* < 0.001) in patients with recurrent OASIs. Hypertension^18^ and diabetes^30^ were analyzed by only one study, without evidence of any association with the considered outcome.

### 3.5 Neonatal characteristics

Neonatal characteristics included weight, difference in weight compared with index pregnancy, gender, cranial circumference. Neonatal weight was considered by 14 studies.^9^, ^18^, ^19^, ^20^, ^21^, ^22^, ^24^, ^25^, ^26^, ^27^, ^28^, ^29^, ^30^, ^31^ Data pooling was possible for four of them.^9^, ^19^, ^21^, ^26^ The meta‐analysis did not show any relationship with OASI recurrence. One study^21^ evaluated the difference in weight between the newborn in the index pregnancy and in the subsequent delivery, finding that the birth weight of subsequent neonates of women who did not sustain a recurrent severe tear was significantly lower than that of their previous child. Head circumference was not associated with the risk of recurrent OASI according to data pooling carried on two studies.^19^, ^26^ The role of neonatal gender was considered by two papers,^18^, ^19^ but no relationship was found with the risk of OASI recurrence.

### 3.6 Others

These included maternal unit deliveries per year and impact of the father. Both were considered by only one study. According to Baghestan et al.,^20^ maternity units with over 3000 deliveries per year were associated with a higher recurrence of OASI in the second delivery, with an adjusted odds ratio of 1.40 (confidence interval 1.20–1.80). The same study reported that a man who fathered a birth resulting in an OASI was more likely to father a subsequent birth resulting in an OASI in another woman who gave birth in the same maternity unit, with an adjusted odds ratio of 2.10 (confidence interval 1.20–3.70).

## 4 DISCUSSION

Currently, the Royal College of Obstetricians and Gynaecologists recommends counseling all women who have suffered from an OASI regarding the mode of delivery in the subsequent pregnancy.^32^ According to the study considered by these guidelines, asymptomatic women with negligible abnormality at anorectal manometry and endoanal ultrasonography can safely undergo vaginal delivery without significant deterioration in anal sphincter function or quality of life.^33^ However, the absolute incidence of OASI recurrence is higher than the primary event, and the risk factors involved in OASI recurrence in women admitted to vaginal delivery are not defined. This systematic review aimed to evaluate the risk factors for recurrent OASIs and describe the impact of risk factors proposed by literature, in terms of significance and strength of association. Our meta‐analysis of 15 studies involving 697 082 women showed that maternal age, gestational age, occiput posterior presentation, oxytocin augmentation, operative delivery, and shoulder dystocia are associated with the risk of recurrent OASI in the subsequent delivery. Notably, episiotomy was not shown to be protective towards sphincter tear recurrence.

The major strengths of our analysis are the robust methodology and the large population considered. Moreover, some of the studies analyzed in the review relied on diagnostic coding from databases and registries, which can be considered accurate. However, there are certain limitations that should be stated. First, different obstetric practices may affect the results. Midwife and physician experience as well as population characteristics may act as confounding factors. Moreover, there is a lack of data about protocols for specific and relevant obstetric practice. For instance, data on specific indications for episiotomy were not available. Similar considerations can be made about the angle and the type of episiotomy, as in most studies the type of episiotomy performed (mediolateral versus midline) was not available. The second point is the high heterogeneity among the studies’ designs and outcomes measures, which leads to the fact that we could only merge data for a few of them. Some risk factors were available for just one study and cannot be meta‐analyzed. Others that were potentially relevant were not even considered in the given studies reported and, hence, could not be commented on, including midwife experience and the woman's position during delivery.^6^, ^34^ Moreover, none of the studies investigated the factors involved in the Royal College of Obstetricians and Gynaecologists’ recommendation for mode of delivery counseling, namely patients’ symptoms, endoanal ultrasonography, and anorectal manometry findings.^33^

The third point is that the papers investigated the impact of individual risk factors, but little information was available about the association between them. Jangö et al.^26^ reported that almost half of patients with OASI recurrence have two or more risk factors. Similar considerations can be made for risk factors identified by this review. For instance, shoulder dystocia can be associated with both vacuum extraction and oxytocin augmentation, and also with advanced gestational age and advanced maternal age.^35^ As a consequence, it is to be determined if the combination of individual risk factors is cumulative. Moreover, due to the low number of studies pooled, it was not possible to properly assess the presence of publication bias in the meta‐analyses performed, except for those for episiotomy and operative delivery. In these two cases, no evidence of publication bias was detectable according to the funnel plot and Egger test.

Finally, identified risk factors were either unmodifiable or poorly modifiable, and no protective measures were identified. Only maternal age is known prenatally and available when counseling women about the mode of delivery. Advanced gestational age can also be potentially considered in counseling, but the absolute difference in gestational age between recurrent and non‐recurrent OASIs is probably clinically non‐significant. The other risk factors, which include oxytocin augmentation, instrumental delivery, occiput posterior position, and shoulder dystocia, develop intrapartum, and very little or nothing can be done to prevent them. These limitations are similar to those applicable to models developed in the last few years to predict and prevent primary OASI.^35^, ^36^ These statistical models are based on risk factors that are either unmodifiable or poorly modifiable, thus failing to be useful in the prevention of OASI during vaginal birth. Our comprehension of the pathogenesis and prevention of both the primary event and the recurrence is still far from being fully understood. This confirms that proper counseling is of the utmost importance before admitting patients to vaginal delivery after OASI, as there are no effective intrapartum measures, including prophylactic episiotomy, to reduce the risk of recurrence. Elective cesarean section is the only effective means to eliminate the risk of recurrent sphincter injury. As a consequence, OASI in the first delivery involves an increased risk of an elective cesarean section in the subsequent delivery; 22% of consultants in the UK would recommend an elective cesarean section to prevent anal incontinence.^37^, ^38^ However, 2.3 cesarean sections are estimated to be needed in order to prevent one case of anal incontinence, and this comes at the price of a higher morbidity (11.3% versus 4.2%) compared with vaginal delivery.^39^ All of these elements should be taken into account when counseling women with a previous OASI in order to obtain adequate informed consent about the mode of delivery.

## 5 CONCLUSION

The meta‐analysis showed that maternal age, gestational age, occiput posterior presentation, oxytocin augmentation, operative delivery, and shoulder dystocia are associated with the risk of recurrent OASIs in the subsequent delivery. Episiotomy is not protective and should only be performed if clinically indicated.

## CONFLICTS OF INTEREST

The authors have no conflicts of interest.

## AUTHOR CONTRIBUTIONS

All authors were responsible for project development, data collection, data analysis, and manuscript writing.

## Supporting information

Fig S1

Fig S2

Appendix S1

File S1

File S2
