# Supplementary figures and images for: Risk factors for obstetric anal sphincter injury recurrence: A systematic review and meta‐analysis
Source: Int J Gynaecol Obstet. 2021 Oct 20;158(1):27–34. doi: 10.1002/ijgo.13950 (PMC9298380; doi:10.1002/ijgo.13950)

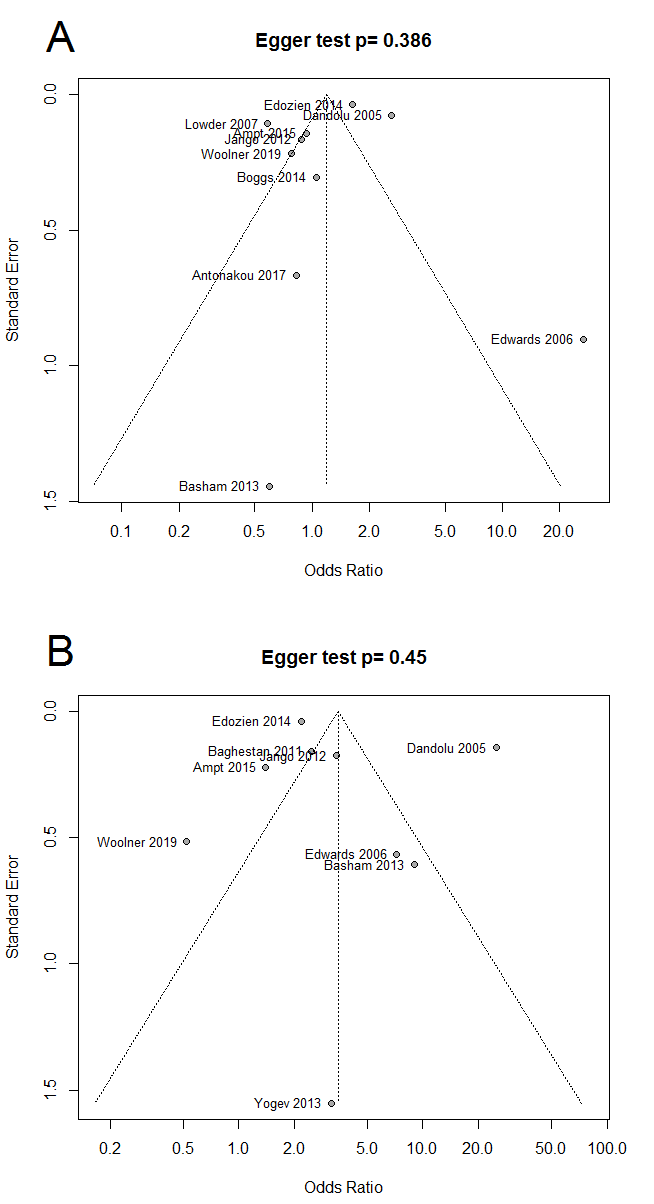

Supplement: Supplementary file 1 — Fig S1 [file IJGO-158-27-s001.tiff]

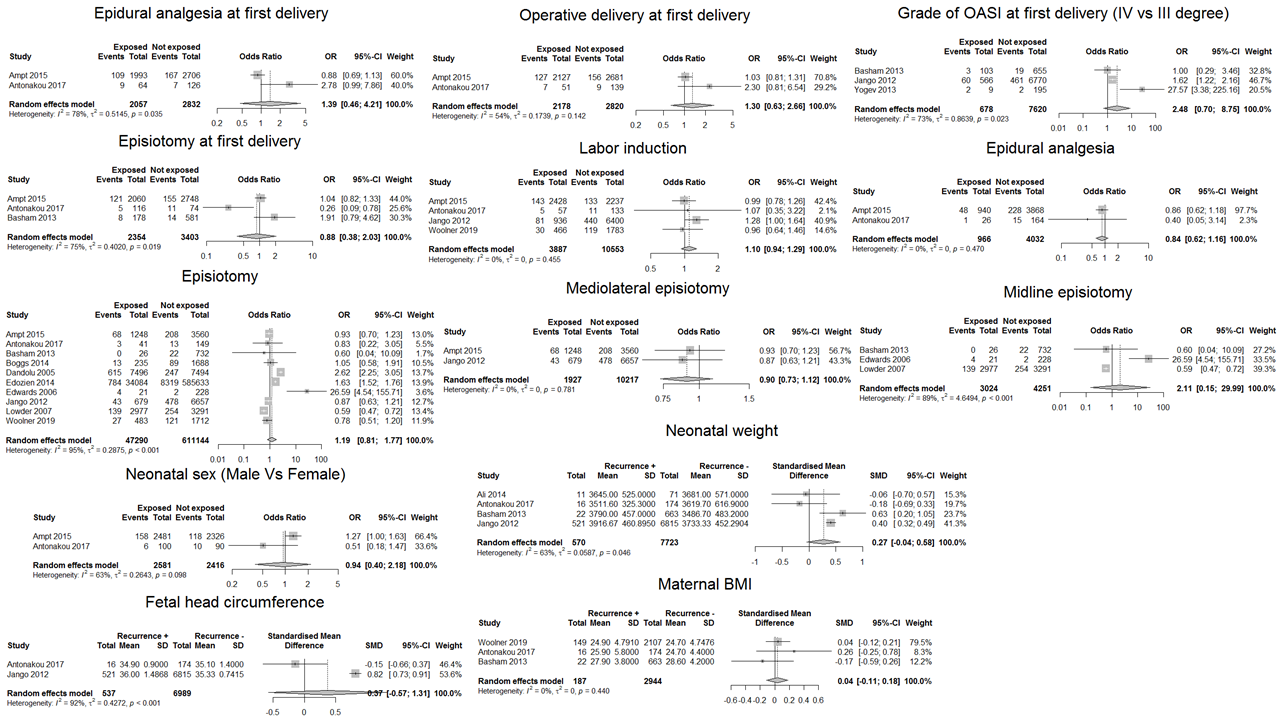

Supplement: Supplementary file 2 — Fig S2 [file IJGO-158-27-s004.tiff]
