# Supplementary material for: Risk factors for obstetric anal sphincter injury recurrence: A systematic review and meta‐analysis
Source: Int J Gynaecol Obstet. 2021 Oct 20;158(1):27–34. doi: 10.1002/ijgo.13950 (PMC9298380; doi:10.1002/ijgo.13950)
Supplement: Supplementary file 3 — Appendix S1 [file IJGO-158-27-s002.docx]

**Appendix S1**. Search strategy.

|  | OASIS AND subsequent pregnancies 26 |
| --- | --- |
| [#1](http://www.ncbi.nlm.nih.gov/pubmed/advanced) | ("poly(divinyl-co-N-vinylpyrrolidinone)"[Supplementary Concept] OR "poly(divinyl-co-N-vinylpyrrolidinone)"[All Fields] OR "oasis"[All Fields]) AND (subsequent[All Fields] AND ("pregnancy"[MeSH Terms] OR "pregnancy"[All Fields] OR "pregnancies"[All Fields])) |
|  | OASIS AND future pregnancies 14 |
| #2 | ("poly(divinyl-co-N-vinylpyrrolidinone)"[Supplementary Concept] OR "poly(divinyl-co-N-vinylpyrrolidinone)"[All Fields] OR "oasis"[All Fields]) AND (("forecasting"[MeSH Terms] OR "forecasting"[All Fields] OR "future"[All Fields]) AND ("pregnancy"[MeSH Terms] OR "pregnancy"[All Fields] OR "pregnancies"[All Fields])) |
|  | OASIS AND recurrence 29 |
| #3 | ("poly(divinyl-co-N-vinylpyrrolidinone)"[Supplementary Concept] OR "poly(divinyl-co-N-vinylpyrrolidinone)"[All Fields] OR "oasis"[All Fields]) AND ("recurrence"[MeSH Terms] OR "recurrence"[All Fields]) |
|  | OASIS AND risk factors 185 |
| #4 | ("poly(divinyl-co-N-vinylpyrrolidinone)"[Supplementary Concept] OR "poly(divinyl-co-N-vinylpyrrolidinone)"[All Fields] OR "oasis"[All Fields]) AND ("risk factors"[MeSH Terms] OR ("risk"[All Fields] AND "factors"[All Fields]) OR "risk factors"[All Fields]) |
|  | anal sphincter injuries AND subsequent pregnancies 82 |
| #5 | (("anal canal"[MeSH Terms] OR ("anal"[All Fields] AND "canal"[All Fields]) OR "anal canal"[All Fields] OR ("anal"[All Fields] AND "sphincter"[All Fields]) OR "anal sphincter"[All Fields]) AND ("injuries"[Subheading] OR "injuries"[All Fields] OR "wounds and injuries"[MeSH Terms] OR ("wounds"[All Fields] AND "injuries"[All Fields]) OR "wounds and injuries"[All Fields])) AND (subsequent[All Fields] AND ("pregnancy"[MeSH Terms] OR "pregnancy"[All Fields] OR "pregnancies"[All Fields])) |
|  | anal sphincter injuries AND future pregnancies 37 |
| #6 | (("anal canal"[MeSH Terms] OR ("anal"[All Fields] AND "canal"[All Fields]) OR "anal canal"[All Fields] OR ("anal"[All Fields] AND "sphincter"[All Fields]) OR "anal sphincter"[All Fields]) AND ("injuries"[Subheading] OR "injuries"[All Fields] OR "wounds and injuries"[MeSH Terms] OR ("wounds"[All Fields] AND "injuries"[All Fields]) OR "wounds and injuries"[All Fields])) AND (("forecasting"[MeSH Terms] OR "forecasting"[All Fields] OR "future"[All Fields]) AND ("pregnancy"[MeSH Terms] OR "pregnancy"[All Fields] OR "pregnancies"[All Fields])) |
|  | anal sphincter injuries AND recurrence 68 |
| #7 | (("anal canal"[MeSH Terms] OR ("anal"[All Fields] AND "canal"[All Fields]) OR "anal canal"[All Fields] OR ("anal"[All Fields] AND "sphincter"[All Fields]) OR "anal sphincter"[All Fields]) AND ("injuries"[Subheading] OR "injuries"[All Fields] OR "wounds and injuries"[MeSH Terms] OR ("wounds"[All Fields] AND "injuries"[All Fields]) OR "wounds and injuries"[All Fields])) AND ("recurrence"[MeSH Terms] OR "recurrence"[All Fields]) |
|  | anal sphincter injuries AND risk factors 420 |
| #8 | (("anal canal"[MeSH Terms] OR ("anal"[All Fields] AND "canal"[All Fields]) OR "anal canal"[All Fields] OR ("anal"[All Fields] AND "sphincter"[All Fields]) OR "anal sphincter"[All Fields]) AND ("injuries"[Subheading] OR "injuries"[All Fields] OR "wounds and injuries"[MeSH Terms] OR ("wounds"[All Fields] AND "injuries"[All Fields]) OR "wounds and injuries"[All Fields])) AND ("risk factors"[MeSH Terms] OR ("risk"[All Fields] AND "factors"[All Fields]) OR "risk factors"[All Fields]) |
|  | anal sphincter tears AND subsequent pregnancies 23 |
| #9 | (("anal canal"[MeSH Terms] OR ("anal"[All Fields] AND "canal"[All Fields]) OR "anal canal"[All Fields] OR ("anal"[All Fields] AND "sphincter"[All Fields]) OR "anal sphincter"[All Fields]) AND ("tears"[MeSH Terms] OR "tears"[All Fields])) AND (subsequent[All Fields] AND ("pregnancy"[MeSH Terms] OR "pregnancy"[All Fields] OR "pregnancies"[All Fields])) |
|  | anal sphincter tears AND future pregnancies 13 |
| #10 | (("anal canal"[MeSH Terms] OR ("anal"[All Fields] AND "canal"[All Fields]) OR "anal canal"[All Fields] OR ("anal"[All Fields] AND "sphincter"[All Fields]) OR "anal sphincter"[All Fields]) AND ("tears"[MeSH Terms] OR "tears"[All Fields])) AND (("forecasting"[MeSH Terms] OR "forecasting"[All Fields] OR "future"[All Fields]) AND ("pregnancy"[MeSH Terms] OR "pregnancy"[All Fields] OR "pregnancies"[All Fields])) |
|  | anal sphincter tears AND recurrence 10 |
| #11 | (("anal canal"[MeSH Terms] OR ("anal"[All Fields] AND "canal"[All Fields]) OR "anal canal"[All Fields] OR ("anal"[All Fields] AND "sphincter"[All Fields]) OR "anal sphincter"[All Fields]) AND ("tears"[MeSH Terms] OR "tears"[All Fields])) AND ("recurrence"[MeSH Terms] OR "recurrence"[All Fields]) |
|  | anal sphincter tears AND risk factors 130 |
| #12 | (("anal canal"[MeSH Terms] OR ("anal"[All Fields] AND "canal"[All Fields]) OR "anal canal"[All Fields] OR ("anal"[All Fields] AND "sphincter"[All Fields]) OR "anal sphincter"[All Fields]) AND ("tears"[MeSH Terms] OR "tears"[All Fields])) AND ("risk factors"[MeSH Terms] OR ("risk"[All Fields] AND "factors"[All Fields]) OR "risk factors"[All Fields]) |
|  | severe obstetrical tears AND subsequent pregnancies 10 |
| #13 | (severe[All Fields] AND ("obstetrics"[MeSH Terms] OR "obstetrics"[All Fields] OR "obstetrical"[All Fields]) AND ("tears"[MeSH Terms] OR "tears"[All Fields])) AND (subsequent[All Fields] AND ("pregnancy"[MeSH Terms] OR "pregnancy"[All Fields] OR "pregnancies"[All Fields])) |
|  | severe obstetrical tears AND future pregnancies 3 |
| #14 | (severe[All Fields] AND ("obstetrics"[MeSH Terms] OR "obstetrics"[All Fields] OR "obstetrical"[All Fields]) AND ("tears"[MeSH Terms] OR "tears"[All Fields])) AND (("forecasting"[MeSH Terms] OR "forecasting"[All Fields] OR "future"[All Fields]) AND ("pregnancy"[MeSH Terms] OR "pregnancy"[All Fields] OR "pregnancies"[All Fields])) |
|  | severe obstetrical tears AND recurrence 4 |
| #15 | (severe[All Fields] AND ("obstetrics"[MeSH Terms] OR "obstetrics"[All Fields] OR "obstetrical"[All Fields]) AND ("tears"[MeSH Terms] OR "tears"[All Fields])) AND ("recurrence"[MeSH Terms] OR "recurrence"[All Fields]) |
|  | severe obstetrical tears AND risk factors 57 |
| #16 | (severe[All Fields] AND ("obstetrics"[MeSH Terms] OR "obstetrics"[All Fields] OR "obstetrical"[All Fields]) AND ("tears"[MeSH Terms] OR "tears"[All Fields])) AND ("risk factors"[MeSH Terms] OR ("risk"[All Fields] AND "factors"[All Fields]) OR "risk factors"[All Fields]) |
|  | third degree tears AND subsequent pregnancies 27 |
| #17 | (third[All Fields] AND degree[All Fields] AND ("tears"[MeSH Terms] OR "tears"[All Fields])) AND (subsequent[All Fields] AND ("pregnancy"[MeSH Terms] OR "pregnancy"[All Fields] OR "pregnancies"[All Fields])) |
|  | third degree tears AND future pregnancies 12 |
| #18 | (third[All Fields] AND degree[All Fields] AND ("tears"[MeSH Terms] OR "tears"[All Fields])) AND (("forecasting"[MeSH Terms] OR "forecasting"[All Fields] OR "future"[All Fields]) AND ("pregnancy"[MeSH Terms] OR "pregnancy"[All Fields] OR "pregnancies"[All Fields])) |
|  | third degree tears AND recurrence 10 |
| #19 | (third[All Fields] AND degree[All Fields] AND ("tears"[MeSH Terms] OR "tears"[All Fields])) AND ("recurrence"[MeSH Terms] OR "recurrence"[All Fields]) |
|  | third degree tears AND risk factors 124 |
| #20 | (third[All Fields] AND degree[All Fields] AND ("tears"[MeSH Terms] OR "tears"[All Fields])) AND ("risk factors"[MeSH Terms] OR ("risk"[All Fields] AND "factors"[All Fields]) OR "risk factors"[All Fields]) |
|  | fourth degree tears AND subsequent pregnancies 18 |
| #21 | (fourth[All Fields] AND degree[All Fields] AND ("tears"[MeSH Terms] OR "tears"[All Fields])) AND (subsequent[All Fields] AND ("pregnancy"[MeSH Terms] OR "pregnancy"[All Fields] OR "pregnancies"[All Fields])) |
|  | fourth degree tears AND future pregnancies 7 |
| #22 | (fourth[All Fields] AND degree[All Fields] AND ("tears"[MeSH Terms] OR "tears"[All Fields])) AND (("forecasting"[MeSH Terms] OR "forecasting"[All Fields] OR "future"[All Fields]) AND ("pregnancy"[MeSH Terms] OR "pregnancy"[All Fields] OR "pregnancies"[All Fields])) |
|  | fourth degree tears AND recurrence 6 |
| #23 | (fourth[All Fields] AND degree[All Fields] AND ("tears"[MeSH Terms] OR "tears"[All Fields])) AND ("recurrence"[MeSH Terms] OR "recurrence"[All Fields]) |
|  | fourth degree tears AND risk factors 77 |
| #24 | (fourth[All Fields] AND degree[All Fields] AND ("tears"[MeSH Terms] OR "tears"[All Fields])) AND ("risk factors"[MeSH Terms] OR ("risk"[All Fields] AND "factors"[All Fields]) OR "risk factors"[All Fields]) |
