# Supplementary material for: Risk factors for obstetric anal sphincter injury recurrence: A systematic review and meta‐analysis
Source: Int J Gynaecol Obstet. 2021 Oct 20;158(1):27–34. doi: 10.1002/ijgo.13950 (PMC9298380; doi:10.1002/ijgo.13950)
Supplement: Supplementary file 5 — File S2 [file IJGO-158-27-s005.pdf]

MOOSE Guidelines for Meta-Analyses and Systematic Reviews of Observational Studies*

	Topic	Page numbe
Title	Identify the study as a meta-analysis (or systematic review)	1
Abstract	Use the journal's structured format	2
Introduction	**Present:**	
	The clinical problem	4
	The hypothesis	4
	A statement of objectives that includes the study population, the condition of interest, the exposure or intervention, and the outcome(s) considered	4
Sources	**Describe:**	
	Qualifications of searchers (eg, librarians and investigators)	5
	Search strategy, including time period included in the synthesis and keywords	5
	Effort to include all available studies, including contact with authors	5
	Databases and registries searched	5-6
	Search software used, name and version, including special features used (e.g. explosion)	5-6
	Use of hand searching (e.g, reference lists of obtained articles)	5-6
	List of citations located and those excluded, including justification	5-6
	Method of addressing articles published in languages other than English	5-6
	Method of handling abstracts and unpublished studies	5-6
	Description of any contact with authors	5-6
Study Selection	**Describe**	
	Types of study designs considered	5-6
	Relevance or appropriateness of studies gathered for assessing the hypothesis to be tested	5-6
	Rationale for the selection and coding of data (eg, sound clinical principles or convenience)	5-6
	Documentation of how data were classified and coded (eg, multiple raters, blinding, and inter-rater reliability)	5-6
	Assessment of confounding (e.g. comparability of cases and controls in studies where appropriate)	6-7
	Assessment of study quality, including blinding of quality assessors; stratification or regression on possible predictors of study results	6-7
	Assessment of heterogeneity	6-7
	Statistical methods (eg, complete description of fixed or random effects models, justification of whether the chosen models account for predictors of study results, dose-response models, or cumulative meta-analysis) in sufficient detail to be replicated	6-7
Results	**Present**	
	A graph summarizing individual study estimates and the overall estimate	7-11
	A table giving descriptive information for each included study	7-11
	Results of sensitivity testing (eg, subgroup analysis)	7-11
	Indication of statistical uncertainty of findings	7-11
Discussion	**Discuss**	
	Strengths and weaknesses	11-14
	Potential biases in the review process (eg, publication bias)	11-14

	Assessment of quality of included studies	11-14
	Consideration of alternative explanations for observed results	11-14
	Generalization of the conclusions (ie, appropriate for the data presented and within the domain of the literature review)	11-14
	Guidelines for future research	11-14
	Disclosure of funding source	11-14

*Modified from Stroup DF, Berlin JA, Morton SC, Olkin I, Williamson GD, Rennie D, et al. Meta-analysis of observational studies in epidemiology: a proposal for reporting. Meta-analysis Of Observational Studies in Epidemiology (MOOSE) group. JAMA 2000;283:2008–12. Copyrighted © 2000, American Medical Association. All rights reserved.
